# Supplementary figures and images for: A real data-driven simulation strategy to select an imputation method for mixed-type trait data
Source: PLoS Comput Biol. 2023 Mar 22;19(3):e1010154. doi: 10.1371/journal.pcbi.1010154 (PMC10069776; doi:10.1371/journal.pcbi.1010154)

a)

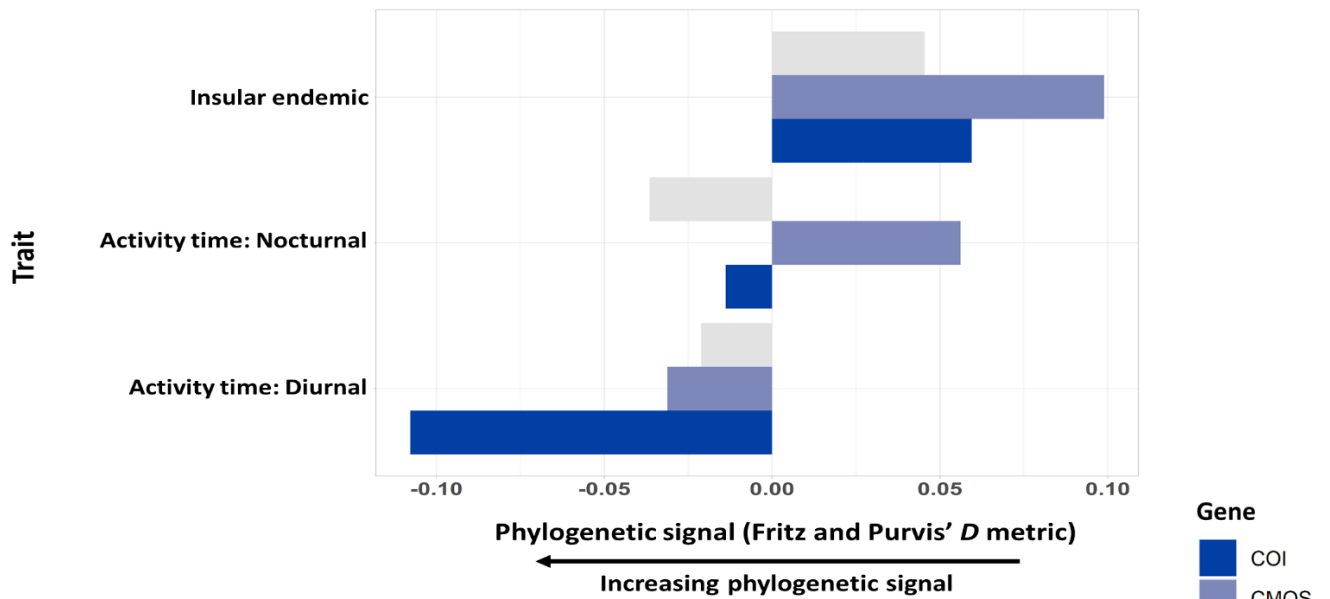

b)

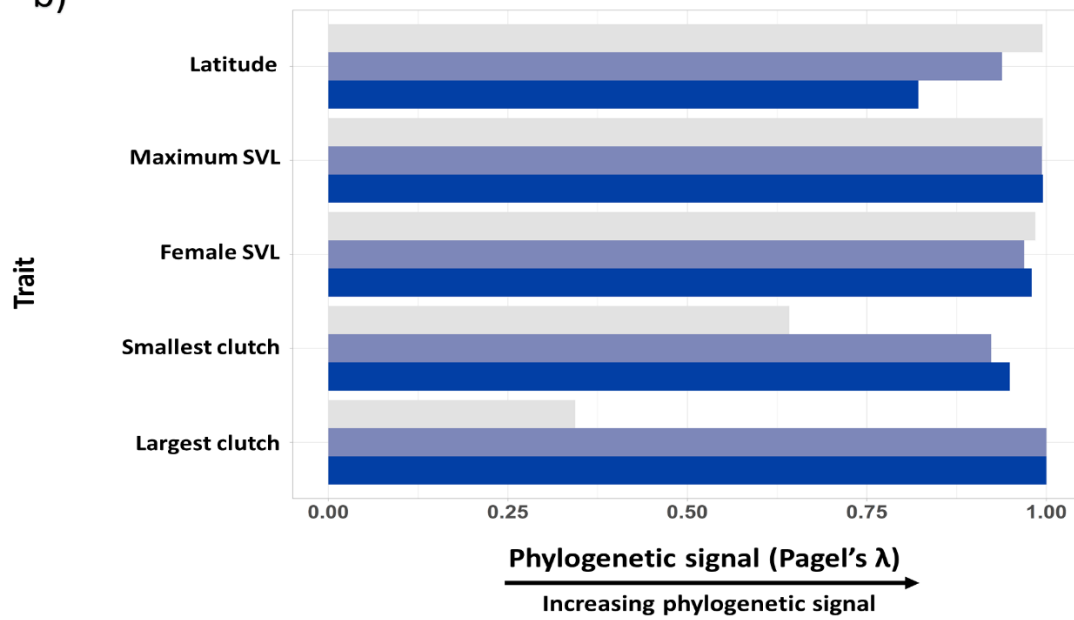

Supplement: S1 Fig — Measures of phylogenetic signal for a) categorical and b) numerical traits in gene trees constructed for mitochondrial COI and nuclear c-mos and RAG1. Asterisks indicate significance at the 0.05 level, according to results from hypothesis tests comparing the results to a null model (no phylogenetic signal). Fritz and Purvis’ D metric [41] and Pagel’s λ [42] were used to measure phylogenetic signal for categorical and numerical traits, respectively. In the case of D, lower values are indicative of higher levels of phylogenetic conservation for the trait; conversely, higher values of λ suggest stronger phylogenetic signal. As the D metric only measures the phylogenetic signal of binary traits, the three-level categorical trait AT was broken down into the binary traits “AT: Diurnal” and “AT: Nocturnal”. (PDF) [file pcbi.1010154.s002.pdf]
