# Supplementary material for: A real data-driven simulation strategy to select an imputation method for mixed-type trait data
Source: PLoS Comput Biol. 2023 Mar 22;19(3):e1010154. doi: 10.1371/journal.pcbi.1010154 (PMC10069776; doi:10.1371/journal.pcbi.1010154)
Supplement: S1 Table — (PDF) [file pcbi.1010154.s003.pdf]

S1 Table. Sequence identifiers.

Species name	COI Process ID	c-mos Accession Number	RAG1 Accession Number
Acanthodactylus boskianus	NPLRP211-08	EF632251	EF632206
Aeluroscalabotes felinus	GBMNA11757-19	HQ426517	JN654855
Agama agama	BACOR025-13	AF137530	EU402825
Agamura persica	ABLRP470-07	DQ852728	JQ945281
Alsophylax pipiens	NPLRP398-08	JQ945531	JQ945284
Anniella pulchra	EANAH930-12	AY487350	AY662605
Apathya cappadocica	GBGC12728-13	EF632268	EF632223
Aprasia parapulchella	GBMTG4679-16	AY134539	HQ426260
Bipes biporus	GBMTG502-16	AF039482	AY662616
Bipes canaliculatus	GBMNA11783-19	FJ518700	FJ518701
Blaesodactylus antongilensis	REPT235-12	JQ945534	EU054230
Blanus cinereus	GBMTG1541-16	DQ324864	EU108523
Brookesia ambreensis	REPT049-12	FJ984313	FJ984243
Brookesia antakarana	REPT050-12	FJ984312	FJ984242
Brookesia brygooi	REPT153-12	FJ984306	FJ984236
Brookesia decaryi	GBMNA11833-19	FJ984308	FJ984238
Brookesia ebenaui	REPT057-12	FJ984300	FJ984230
Brookesia exarmata	REPT360-12	FJ984291	FJ984220
Brookesia griveaudi	REPT371-12	FJ984321	FJ984251
Brookesia minima	REPT396-12	FJ984280	FJ984209
Brookesia peyrierasi	REPT236-12	FJ984287	FJ984216
Brookesia stumpffi	REPT051-12	FJ984318	FJ984247
Brookesia superciliaris	GBGC6845-09	FJ984303	FJ984233
Brookesia tuberculata	REPT052-12	FJ984281	FJ984210
Bunopus tuberculatus	NPLRP073-08	AF148706	JQ945287
Caledoniscincus austrocaledonicus	GBGCR3727-19	DQ675404	EU568024
Calumma gastrotaenia	REPT251-12	FJ984263	FJ984191
Calumma nasutum	REPT233-12	JN030463	HQ130637

Chalarodon madagascariensis	GBMNA11850-19	AY987987	FJ356745
Chlamydosaurus kingii	GBMNA18857-19	DQ340665	JF806191
Cnemaspis limi	GBMNA11758-19	EF534935	EF534809
Coleonyx variegatus	GBMTG991-16	EF534901	EU108526
Crossobamon orientalis	ABLRP217-07	DQ852730	JQ945299
Crotaphytus collaris	EANAH716-12	AY987985	FJ356749
Cyrtodactylus irregularis	GBGCR6135-19	JQ945551	JQ945302
Cyrtopodion scabrum	DJIB114-17	HQ426532	HQ426275
Dibamus novaeguineae	GBGC9701-09	EF450999	EU108529
Dipsosaurus dorsalis	EANAH605-12	AF148705	FJ356747
Ebenavia inunguis	BACOR034-13	FJ830144	EF536143
Eremias arguta	NPLRP542-08	EF632258	EF632213
Eublepharis macularius	GBGCR6235-19	EU366458	EF534776
Eutropis multifasciata	IQHLM132-06	DQ238978	AY444055
Furcifer lateralis	REPT394-12	FJ984272	JQ073208
Furcifer polleni	BACOR067-13	FJ984267	JQ073215
Furcifer willsii	REPT250-12	HQ130551	HQ130640
Geckolepis maculata	BACOR081-13	JQ945562	EU054211
Gehyra mutilata	NPRPV022-08	FJ830146	FJ830237
Gekko chinensis	GBMNA11763-19	JQ945571	JN019123
Gekko gecko	NPRPV009-08	EU366455	AY662625
Gekko vittatus	GBGC10139-09	JQ945575	JN019137
Gonatodes albogularis	GBGCR6260-19	EF564078	EF534797
Goniurosaurus luii	GBMNA11755-19	HQ426538	HQ426287
Heloderma suspectum	GBGCR449-15	AY662566	AY662606
Hemidactylus flaviviridis	DJIB124-17	HQ426541	HM559694
Hemidactylus frenatus	GBGC10802-13	EF534940	EF534814
Hemidactylus mercatorius	BACOR085-13	AY863046	JQ073244
Hemidactylus platycephalus	BACOR077-13	AY863045	JQ073246
Hemidactylus platyurus	NPRPV024-08	HQ426530	HM559685

Hemidactylus robustus	DJIB231-17	HQ426549	EU054271
Hemitheconyx caudicinctus	GBMNA11756-19	HQ426552	HQ426294
Hemitheconyx taylori	GBGC11800-13	HQ426553	HQ426295
Heteronotia binoei	GBGC4418-08	JQ945580	EU054285
Homonota fasciata	GBMNA18955-19	EU293674	EU293629
Lacerta agilis	FBHER281-14	EU365405	EF632222
Lacerta viridis	GBMTG853-16	DQ097131	EU108535
Lampropholis guichenoti	GBGCR3728-19	DQ675352	EU568111
Leiolepis belliana	GBGCR211-15	FJ984253	AY662587
Lepidodactylus lugubris	GBMNA11768-19	EF534938	EF534812
Lepidophyma flavimaculatum	GBMTG982-16	EU116715	EU108567
Lygodactylus miops	REPT023-12	HQ426556	HQ426299
Lygodactylus mirabilis	REPT385-12	HQ426557	HQ426300
Madascincus melanopleura	REPT223-12	AY802768	HM161147
Marmorosphax tricolor	GBGCR3729-19	DQ675367	EU568023
Matoatoa brevipes	REPT295-12	JQ945587	EF490724
Mesalina guttulata	NPLRP518-08	EF632274	EF632231
Morethia adelaidensis	GBGCR3730-19	DQ675368	EU568109
Nannoscincus mariei	GBGCR3731-19	DQ675372	EU568021
Oligosoma microlepis	GBGCR3874-19	DQ675375	EU568088
Oligosoma smithi	GBGCR3978-19	DQ675386	EU568090
Oligosoma suteri	GBGCR3996-19	DQ675387	EU568106
Oligosoma zelandicum	GBGCR4026-19	AY818781	EU568082
Oplurus cuvieri	GBGCR1711-18	EU099677	AY662601
Oplurus cyclurus	REPT095-12	EU099680	GU457973
Paragehyra gabriellae	REPT083-12	JQ945603	JQ945328
Paroedura androyensis	REPT383-12	HQ256721	EF490721
Paroedura bastardi	REPT292-12	HQ256725	EF536163
Paroedura gracilis	GBGCR282-15	HQ256726	EF536161
Paroedura lohatsara	GBGCR439-15	HQ256731	EF536155

Paroedura masobe	GBGCR440-15	HQ426560	EF536145
Paroedura oviceps	GBGCR441-15	HQ256733	EF536160
Paroedura picta	GBMNA11769-19	EU293692	EF536150
Paroedura stumpffi	GBGCR6441-19	HQ256740	EF536154
Phelsuma abbotti	REPT364-12	AY221346	FJ830148
Phelsuma antanosy	REPT097-12	FJ830064	FJ830156
Phelsuma barbouri	REPT384-12	FJ830069	FJ830161
Phelsuma berghofi	REPT320-12	FJ830070	FJ830162
Phelsuma breviceps	REPT003-12	FJ830072	FJ830164
Phelsuma dubia	BACOR114-13	FJ830080	FJ830172
Phelsuma guimbeaui	GBMNA18868-19	AY221372	FJ830221
Phelsuma guttata	REPT020-12	FJ830085	FJ830177
Phelsuma hielscheri	REPT121-12	FJ830086	FJ830178
Phelsuma laticauda	BACOR117-13	AY221343	FJ830189
Phelsuma lineata	REPT001-12	AY221342	JN654861
Phelsuma madagascariensis	REPT298-12	EF534937	EF534811
Phelsuma malamakibo	REPT348-12	FJ830108	FJ830200
Phelsuma modesta	REPT285-12	FJ830110	FJ830202
Phelsuma mutabilis	REPT352-12	FJ830114	FJ830206
Phelsuma pusilla	REPT305-12	FJ830122	FJ830214
Phelsuma quadriocellata	REPT199-12	FJ830123	FJ830215
Phelsuma ravenala	REPT319-12	FJ830078	FJ830170
Phelsuma serraticauda	REPT324-12	FJ830131	FJ830223
Phelsuma standingi	REPT289-12	FJ830133	FJ830225
Phoenicolacerta kulzeri	GBMNA11795-19	GQ142151	GQ142161
Phrynocephalus mystaceus	GBGCR6007-19	AF137527	GQ242268
Phyllodactylus unctus	GBMTG2995-16	FJ662503	HQ426312
Plestiodon skiltonianus	EANAH819-12	AF315396	AY662633
Plica plica	GBGCR5143-19	EF615737	FJ356742
Podarcis muralis	FBHER036-09	EF632282	EF632239

Pogona vitticeps	GBMNA11825-19	DQ340691	JF806200
Polychrus marmoratus	GBMNA11856-19	AY987983	FJ356748
Ptyodactylus guttatus	GBMNA11775-19	EU293681	EU293636
Ptyodactylus hasselquistii	NPLRP357-08	EU293682	EU293637
Quedenfeldtia moerens	GBGCR5605-19	HQ426574	HQ426320
Quedenfeldtia trachyblepharus	GBGCR5607-19	EF534930	EF534804
Rhineura floridana	GBMTG497-16	AY444022	AY662618
Saurodactylus mauritanicus	NPLRP424-08	EU014324	EU014356
Sauromalus ater	EANAH588-12	AF315400	AY662591
Scincella lateralis	EANAH892-12	AY217857	HM161236
Scincus scincus	NPLRP289-08	AY217873	HM161238
Shinisaurus crocodilurus	GBMNA11809-19	AY099976	AY662610
Stenodactylus sthenodactylus	ABLRP285-07	JQ945617	JQ945339
Takydromus amurensis	GBGC11979-13	EF632287	EF632244
Takydromus sexlineatus	GBMNA11799-19	EF632288	EF632245
Tarentola annularis	DJIB068-17	AF363552	DQ275456
Tarentola mauritanica	GBGC12027-13	AF363566	EU293641
Teratoscincus microlepis	GBGCR6026-19	EF534926	EF534800
Teratoscincus przewalskii	GBGCR6022-19	AY662569	AY662624
Teratoscincus roborowskii	ZISPG080-09	EF534925	EF534799
Teratoscincus scincus	GBGCR6037-19	EF534927	EF534801
Tracheloptychus madagascariensis	REPT345-12	DQ100104	JQ073186
Tropidurus hispidus	GBGCR1725-18	AY987984	AY988013
Tropidurus insulanus	GBGCR5141-19	EF615738	EF616390
Tropidurus oreadicus	GBGCR1726-18	EF615739	EF616391
Tropiocolotes tripolitanus	GBMNA11771-19	JQ945623	JQ945343
Uroplatus ebenaui	GBMNA11773-19	JN038097	EF490736
Uroplatus giganteus	REPT053-12	JQ945625	EF490738
Uroplatus guentheri	REPT279-12	JQ945626	EF490725

Uta stansburiana	GBMNA11855-19	AF315389	DQ385422
Varanus salvator	GBGCR4034-19	AF435017	EU402828
Xantusia henshawi	EANAH907-12	EU116794	EU108645
Zonosaurus madagascariensis	REPT017-12	DQ100129	JQ073185
Zootoca vivipara	FBHER093-09	EF632292	EF632249

Database identifiers for all sequence records used in this study (n = 152 species). Process IDs are included for cytochrome c oxidase subunit I (COI) sequence records obtained from the Barcode of Life Data (BOLD) System (1). GenBank (2,3) accession numbers are included for nuclear oocyte maturation factor (c-mos) and recombination activating gene 1 (RAG1). c-mos and RAG1 sequences were obtained via the multigene alignment published in Pyron *et al.* (4,5).

References

1. Ratnasingham S, Hebert PDN. BOLD: The Barcode of Life Data System (www.barcodinglife.org). Mol Ecol Notes. 2007;7:355–64.

2. Benson DA, Cavanaugh M, Clark K, Karsch-Mizrachi I, Lipman DJ, Ostell J, et al. GenBank. Nucleic Acids Res. 2012/11/27 ed. 2013 Jan;41(Database issue):D36–42.

3. NCBI Resource Coordinators. Database resources of the National Center for Biotechnology Information. Nucleic Acids Res. 2015/11/28 ed. 2016 Jan 4;44(D1):D7–19.

4. Pyron RA, Burbrink FT, Wiens JJ. A phylogeny and revised classification of Squamata, including 4161 species of lizards and snakes. BMC Evol Biol. 2013 Apr 29;13(1):93.

5. Pyron RA, Burbrink FT, Wiens JJ. Data from: A phylogeny and revised classification of Squamata, including 4161 species of lizards and snakes. Dryad Dataset [Internet]. 2013; Available from: https://doi.org/10.5061/dryad.82h0m
