# Supplementary material for: A real data-driven simulation strategy to select an imputation method for mixed-type trait data
Source: PLoS Comput Biol. 2023 Mar 22;19(3):e1010154. doi: 10.1371/journal.pcbi.1010154 (PMC10069776; doi:10.1371/journal.pcbi.1010154)
Supplement: S2 Table — (PDF) [file pcbi.1010154.s004.pdf]

S2 Table. Taxonomic composition of nearly complete-case trait dataset (*n* = 152).

Family	Common name	Count (N)	Percentage of dataset (%)
Gekkonidae	Common geckos	59	38.8
Chamaeleonidae	Chameleons	17	11.2
Scincidae	Skinks	14	9.2
Lacertidae	Old world runners/Lacertid lizards	11	7.2
Sphaerodactylidae	N/A	8	5.3
Eublepharidae	Eublepharid geckos	6	3.9
Phyllodactylidae	N/A	6	3.9
Agamidae	Old world arboreal lizards/Agamid lizards	5	3.3
Tropiduridae	Tropidurid lizards	4	2.6
Opluridae	N/A	3	2.0
Bipedidae	Two-legged worm lizards	2	1.3
Gerrhosauridae	Plated lizards	2	1.3
Iguanidae	Iguanas	2	1.3
Xantusiidae	Night lizards	2	1.3
Anniellidae	American legless lizards	1	0.7
Blanidae	N/A	1	0.7
Crotaphytidae	Collared lizards/leopard lizards	1	0.7
Dibamidae	N/A	1	0.7
Helodermatidae	Gila monsters	1	0.7
Phrynosomatidae	North American spiny lizards	1	0.7
Polychrotidae	Anoloid lizards	1	0.7
Pygopodidae	Flap-footed lizards	1	0.7
Rhineuridae	North American worm lizards	1	0.7
Shinisauridae	N/A	1	0.7
Varanidae	Monitor lizards	1	0.7

Trait data obtained from Meiri [1,2].

References

1. Meiri S. Traits of lizards of the world: Variation around a successful evolutionary design. Glob Ecol Biogeogr. 2018;27(10):1168–72.

2. Meiri S. Data from: Traits of lizards of the world: Variation around a successful evolutionary design. Dryad Dataset [Internet]. 2019; Available from: https://doi.org/10.5061/dryad.f6t39kj
