# Supplementary material for: A real data-driven simulation strategy to select an imputation method for mixed-type trait data
Source: PLoS Comput Biol. 2023 Mar 22;19(3):e1010154. doi: 10.1371/journal.pcbi.1010154 (PMC10069776; doi:10.1371/journal.pcbi.1010154)
Supplement: S3 Table — (PDF) [file pcbi.1010154.s005.pdf]

S3 Table. Descriptions and additional details for traits in the nearly complete-case dataset.

Trait	Description	Data type	Sample size (*n*)*	Units/Levels (%)	Reference
Activity time	Time of day when species is most active (cathemeral: active at any time (irregular); diurnal: active at day time; nocturnal: active at night time)	Categorical (nominal, multicategorical)	145	Cathemeral (11%)	Meiri [1,2]
				Diurnal (58%)	
				Nocturnal (31%)	
Female snout-vent length	Average female snout-vent length for species	Numerical (continuous)	137	Millimetres (mm)	Meiri [1,2]
Insular endemic	Whether the species lives on an island (Yes) or not (No)	Categorical (nominal, binary)	152	No (54%)	Meiri [1,2]
				Yes (46%)	
Largest clutch	Maximum observed litter size for species	Numerical (count)	142	Eggs/Neonates	Meiri [1,2]
Latitude	Centroid latitude recorded for species	Numerical (continuous)	152	Degrees (°)	Roll *et al.* [3], obtained from Meiri [1,2] dataset
Maximum snout-vent length	Maximum observed snout-vent length for species	Numerical (continuous)	152	Millimetres (mm)	Meiri [1,2]
Smallest clutch	Minimum observed litter size for species	Numerical (count)	141	Eggs/Neonates	Meiri [1,2]

White and gray rows represent numerical and categorical traits, respectively. * = The number of complete observations in the dataset (up to 10% missingness was permitted for each trait).

References

1. Meiri S. Traits of lizards of the world: Variation around a successful evolutionary design. Glob Ecol Biogeogr. 2018;27(10):1168–72.

2. Meiri S. Data from: Traits of lizards of the world: Variation around a successful evolutionary design. Dryad Dataset [Internet]. 2019; Available from: https://doi.org/10.5061/dryad.f6t39kj

3. Roll U, Feldman A, Novosolov M, Allison A, Bauer AM, Bernard R, et al. The global distribution of tetrapods reveals a need for targeted reptile conservation. Nat Ecol Evol. 2017 Nov 1;1(11):1677–82.
